# Mesenchymal stem cell-derived extracellular vesicles may promote breast cancer cell dormancy

**DOI:** 10.1177/2041731418810093

**Published:** 2018-12-25

**Authors:** Jake Casson, Owen G Davies, Carol-Anne Smith, Matthew J Dalby, Catherine C Berry

**Affiliations:** 1 Centre for Cell Engineering, Institute of Molecular, Cell and Systems Biology (IMCSB), The University of Glasgow, Glasgow, UK; 2 School of Sport, Exercise and Health Sciences, Loughborough University, Loughborough, UK

**Keywords:** Breast cancer, mesenchymal stem cell, extracellular vesicle, dormancy, three dimensional

## Abstract

Disseminated breast cancer cells have the capacity to metastasise to the bone marrow and reside in a dormant state within the mesenchymal stem cell niche. Research has focussed on paracrine signalling factors, such as soluble proteins, within the microenvironment. However, it is now clear extracellular vesicles secreted by resident mesenchymal stem cells into this microenvironment also play a key role in the initiation of dormancy. Dormancy encourages reduced cell proliferation and migration, while upregulating cell adhesion, thus retaining the cancer cells within the bone marrow microenvironment. Here, MCF7 breast cancer cells were treated with mesenchymal stem cell–derived extracellular vesicles, resulting in reduced migration in two-dimensional and three-dimensional culture, with reduced cell proliferation and enhanced adhesion, collectively supporting cancer cell dormancy.

## Introduction

Breast cancer is the most prevalent form of malignancy in women.^1^ Dissemination of breast cancer cells (BCCs) to distant sites is believed to be an early event, often occurring before detection of the primary tumour.^2^ More than two-thirds of breast cancers that spread to other parts of the body spread to the bone marrow.^3^ It is now well established that breast cancer recurrence is due to prolonged dormancy within the bone marrow.^4^ This phenomenon is responsible for much of the cancer-associated mortality as metastatic recurrence can occur many years after primary tumour treatment, leading to an uncertainty in the prognosis for patients.^5^

Prior to metastasis, BCCs undergo epithelial–mesenchymal transition (EMT), whereby they lose epithelial traits such as cell adhesion and gain mesenchymal characteristics, becoming migratory.^6,7^ Upon reaching distant secondary sites, such as the bone marrow, a reverse process termed mesenchymal–epithelial transition (MET) then occurs, allowing the BCCs to colonise their secondary microenvironment.^8^ The invading BCCs take advantage of the immune tolerant features and chemotactic properties of resident mesenchymal stem cells (MSCs) and their niche to both promote and support BCC dormancy.^9,10^ In the early stages of metastatic spread, disseminated BCCs undergo an extended period of cycling quiescence in which they are maintained in G_0_/G_1_ phase of the cell cycle.^11^ However, there is a current lack of knowledge of the mechanistic events that allow BCCs to adopt a dormant phenotype in the marrow.^12^ MSCs are thought to interact with invading BCCs during the early stage of entry into the marrow; thus, further study of how these two cell types communicate during the onset of dormancy may allow a deeper understanding of the cellular events involved.^4^

The relationship between marrow MSCs and invading BCCs has to date focussed on more traditional cell-to-cell communication routes, such as paracrine signalling via soluble proteins including cytokines.^13–15^ More recently, attention has shifted towards extracellular vesicles (EVs) as key mediators in cell–cell communication. EVs are small extracellular membrane-enclosed vesicles that contain a variety of molecules including proteins and RNAs.^16–20^ Increasing evidence suggests that interactions between MSCs and tumour cells involve the exchange of information via EVs.^20^ For example, MSC-derived EVs have been reported to contain microRNAs such as miR23b,^21^ miR21 and miR34a,^22^ which have been found to have a tumour-suppressive effect. These EVs also contained tumour-supportive molecules, such as tissue inhibitor of metalloproteases (TIMP)-1 and -2. Within this study, we have shown that MSC-derived EVs have a negative influence on the migration and proliferation of the BCC line MCF7, with an increased adhesion. This suggests a potential role for MSC-EVs in the promotion of BCC MET and perhaps subsequent dormancy.

## Materials and methods

### Expansion cell culture

MCF7 (ATCC) cells were cultured using modified DMEM comprising 400 mL Dulbecco’s modified Eagle’s medium, 100 mL of medium 199, 50 mL of foetal bovine solution, 10 mL penicillin–streptomycin and 5 mL of sodium pyruvate. MCF7 cells were maintained in T75 tissue culture flasks and passaged at approximately 90% confluence using a HEPES saline wash (ThermoFisher) followed by a 3% trypsin/versine solution (ThermoFisher) to remove cells from culture flask. These cells were then centrifuged at 1400 r/min for 4 min and reseeded into new flasks, with media exchanged every 3 days. For EV isolation during MSC culture, foetal bovine serum was centrifuged for 18 h at 120,000g and supernatant was retained to exclude any EVs present.

### EV isolation

MSCs (Promocell) were grown in culture for 1 week using T150 flasks (Corning) to allow the collection of a large volume of culture medium. EVs were then isolated using the ultracentrifugation method used previously16,17 and analysed via dynamic light scattering. Concentration was determined via BCA (ThermoFisher) and FluoroCet (System Biosciences) assays; generating a protein standard then adjusting isolates to the same total protein and measuring fluorescence of acetylcholinesterase (AChE), a known exosomal protein, present within the vesicles. Due to limited supply and assessment of other EV-related studies,^23–25^ the concentration of approximately 2 × 10^7^/mL was used in each experiment.

### Transmission electron microscopy

MSC-derived (Lonza) particles were placed onto Carbon and Formvar coated copper grids from Agar Scientific. Samples were negatively stained with 2% uranyl acetate and imaged with a JEOL 1200 Transmission Electron Microscope with beam voltage of 80 kV and magnification of 200,000×. Images generated were analysed using ImageJ to measure particle area and maximum diameter. In brief, ImageJ was used to trace the outline of the lipid membrane visible on micrographs. Software then calculated the area and maximum diameter by pixel analysis which was then converted to size using the appropriate scaling parameters.

### Generation of three-dimensional MCF7 spheroid cultures

Spheroids were generated using a similar methodology as described in Lewis et al.^25^ Cells were initially seeded at a density of 1 × 10^4^ into a 24-well plate and incubated for 24 h (37°C and 5% CO_2_). After this initial incubation, each well of cells was cultured in a 1 mL suspension of 200 nm diameter, red fluorescently labelled magnetic iron nanoparticles (chemicell – fluidMAG-PEA) in a DMEM solution at a concentration of 0.1 mg nanoparticles/mL of media for 30 min at (37°C and 5% CO_2_) on top of a 24-well magnetic array plate (350 mT magnetic fields). Subsequently, the nanoparticle/DMEM suspension was removed and excess iron washed from each well using HEPES saline. Cells were then detached from the surface using trypsin and resuspended in fresh media in two opposing corners of a six-well plate to prevent disruption of individual magnetic fields. Once in solution, a 13 mm diameter magnet (producing a 350 mT magnetic field) was placed on the top of each well containing cells and incubated for 24 h while the magnetic field draws nanoparticle containing cells together to form a multicellular spheroid. Once formed, the spheroids were carefully transferred into to a liquid type I collagen gel solution, before being allowed to gel, and cultured in 1 mL fresh media.

### Collagen gel preparation

The type I collagen gel for maintaining and culturing spheroids was made up by initially premixing 0.5 mL of foetal bovine solution, 0.5 mL of modified DMEM culture media and 0.5 mL alpha-MEM. Then, 2.5 mL of rat tail collagen (2 mg/mL; First Link UK) was mixed with 1 mL of 0.1 M NaOH, before combining all together. Additional NaOH was then titrated dropwise until entire solution turned from yellow/orange to a stable pink indicating a pH change; 1 mL of this gel solution is used to culture spheroids within a 24-well plate. A setting period of ⩽1 h is permitted prior to the addition of fresh culture media.

### Two-dimensional MCF7 migration

The kit provided by Ibidi allowed the measurement of cell directional movement in response to a chemoattractant. Using the set protocol supplied (in the ‘3D Chemotaxis Assay Using μ-Slide Chemotaxis – 2.2 2D Chemotaxis experiments without Gel’), MCF7s were seeded at a density of 1 × 10^6^. Once both reservoirs were filled with 65 µL chemoattractant-free DMEM, culture medium containing MSC-derived EVs was aspirated into the left reservoir to begin the chemoattraction for the assay. The plate was imaged using a four times objective lens at 120 s intervals over 24 h in a 37°C hot room. Results were then analysed using the ImageJ plugin ‘manual tracking’ and Ibidi’s own ‘Chemotaxis and Migration tool’.

### Three-dimensional MCF7 migration

To better understand MCF7 migratory processes within a bone marrow-like microenvironment, MCF7 (ATCC) spheroids were generated using magnetic nanoparticles and embedded in collagen gels before being treated over 5 days with MSC EV-doped culture medium. Images were captured using Zeiss Axio Vert A1 fluorescent microscope. Diameters were measured using ImageJ.

### Immunocytochemistry

Cells were fixed for 15 min in 4% formaldehyde/PBS solution. Followed by permeabilisation for 5 min at 4°C, blocking for 1 h at 37°C with 1% BSA/PBS solution. Primary antibodies (Ki67 or ALDH1A1; abcam, anti-rabbit) were diluted 1/100 with blocking solution and cells were stained over night at room temperature before washing with 0.1% tween solution five times for 5 min. Secondary antibody was diluted 1/100 with blocking solution with the addition of 1/500 CellTag solution and incubated for 1 h at room temperature. Cells were then visualised on Licor Odyssey SA plate reader and antibody fluorescence normalised to CellTag (Supplementary Figure 1).

### Live/dead assay

100 µL culture medium containing 4 µM ethidium and 2 µM calcein AM (Life Technologies) was added to cells and incubated at 37°C for 30 min. Cells were then washed with 1% PBS before being imaged using Zeiss Axio Vert fluorescence microscope at 20× magnification where cells containing calcein or ethidium were counted.

### Adhesion assay

Monolayers were seeded in a 96-well plate and allowed to attach. A dilution series of MSC-derived EVs was then applied to these for 24 h. Following this period, cells trypsinised and reseeded into a new 96-well plate. These cells were allowed to attach for 30 min before the culture medium was removed. Remaining cells were stained with DAPI for 15 min before being visualised at 20× magnification. The presence of nuclei in 10 random fields was counted across three wells for each MSC EV concentration.

## Results and discussion

### Quantification of MSC-derived EVs isolated from conditioned culture medium

**Figure 1.**
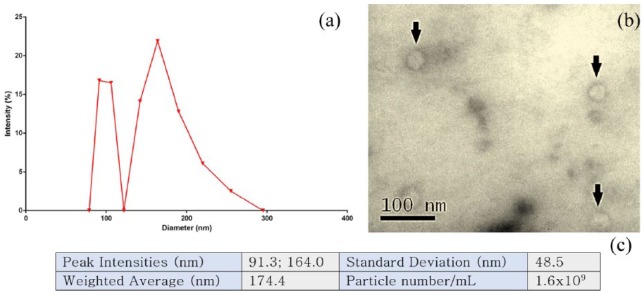
MSC-derived EVs (a) measured using dynamic light scattering and average intensities plotted (n = 3). (b) Transmission electron microscope image of MSC-derived EVs; arrows indicate the presence of vesicles (c) A table detailing key data from dynamic light scatter in addition to particle number acquired through measurement of AChE fluorescence using Fluorocet assay.

EVs isolated from MSC culture medium were quantified using dynamic light scattering and Fluorocet assay. Dynamic light scattering allows the size of particles to be determined (Figure 1(a)) indicating highest peak intensities at 91.3 and 164 nm, with a weighted average diameter of 174.4 nm (Figure 1(b)). This indicates the presence of two vesicle populations: exosomes and larger microvesicles. AChE fluorescence was then assayed (Figure 1(c)) following protein quantification to determine the number of MSC-derived EVs present, found to be 1.6 × 10^9^/mL. The presence of MSC-derived EVs using this method of isolation is indicated by TEM (Figure 1(b)).

### MCF7 cells migrate away from MSC EVs

**Figure 2.**
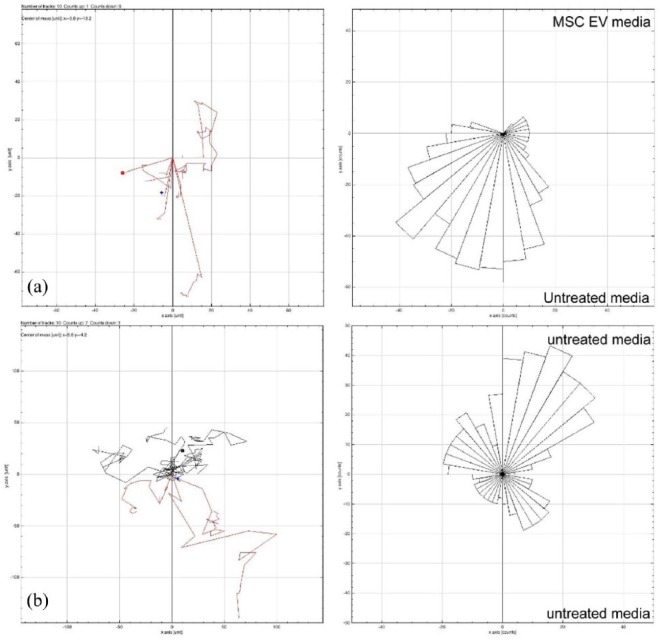
MCF7 cells cultured over 24 h with (a) culture medium containing MSC-derived EVs or (b) control medium, without EVs (10 cells tracked in each condition using ImageJ).

**Figure 3.**
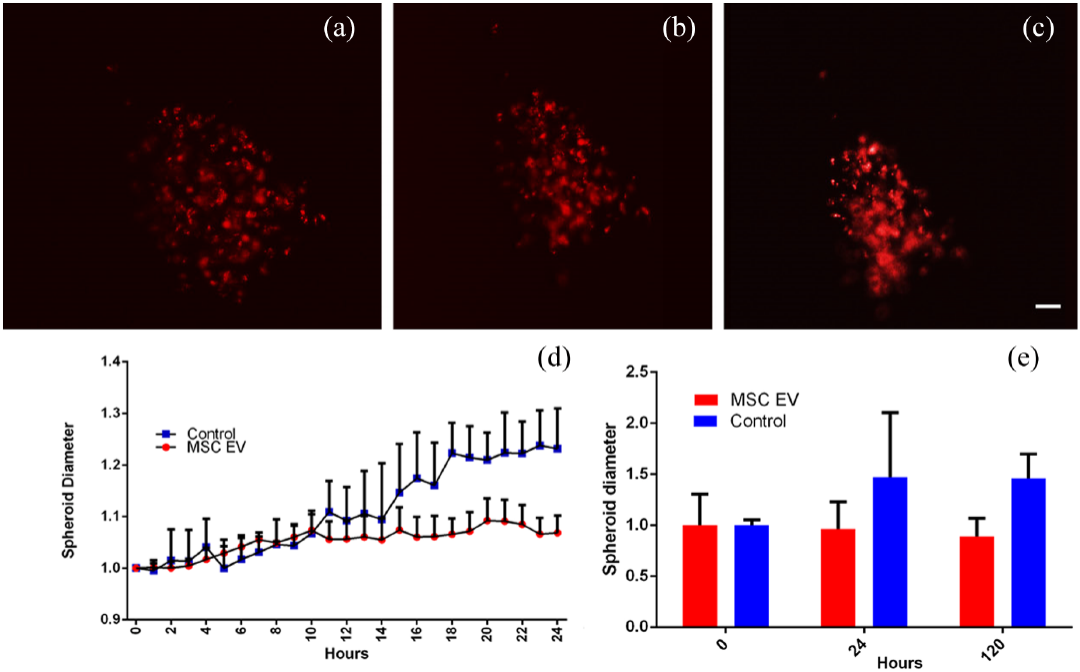
MCF7 spheroid cultured with MSC-derived EVs for (a) 0 h, (b) 24 h and (c) 120 h; note the apparent decrease in diameter; (d) 24-h time lapse of spheroid treated with MSC-derived EVs spheroid diameter normalised to 0 h, n = 3; (e) measurements of further MCF7 spheroids at hours 0, 24 and 120 normalised to 0-h average (n ⩾ 4; scale bar = 10 µm; images recorded using Zeiss Axio Vert A1 microscope).

The effect of MSC-derived EVs on MCF7 cell motility was assessed. Within standard two-dimensional culture, MCF7 cells grown in control culture medium did not migrate preferentially in any direction (Figure 2(b)); however, when treated with cell culture medium containing purified MSC-derived EVs, they become more mobile (Figure 2(a)). Interestingly, they do not move towards the MSC-derived EVs, but appear to migrate away from them. This phenomenon is quite pronounced over 24 h, with no MCF7 cells migrating towards the MSC-derived EVs. This observation therefore appears to support MCF7 cells in monolayer (Figure 2), where the cells are compacting within the spheroids as opposed to migrating out towards the EV stimulus. Over 120 h, mean spheroid area decreases in the presence of MSC-derived EVs (Figure 3(e)).

Consequently, 3D MCF7 cell spheroids were then cultured within a bone marrow-like environment (type I collagen gel) and challenged with MSC-derived EVs. Through measuring the spheroid diameter, it was noted that the spheroids become more compacted over time in response to the EVs when compared to control culture conditions, where instead spheroids increased in diameter by approximately 10% over a 24-h period (Figure 3(a)–(e)). Using a two-way analysis of variance (ANOVA) the difference in spheroid size is significant (p = 0.0011).

### MSC EVs cause decreased proliferation and increase adhesion of MCF7 cells

**Figure 4.**
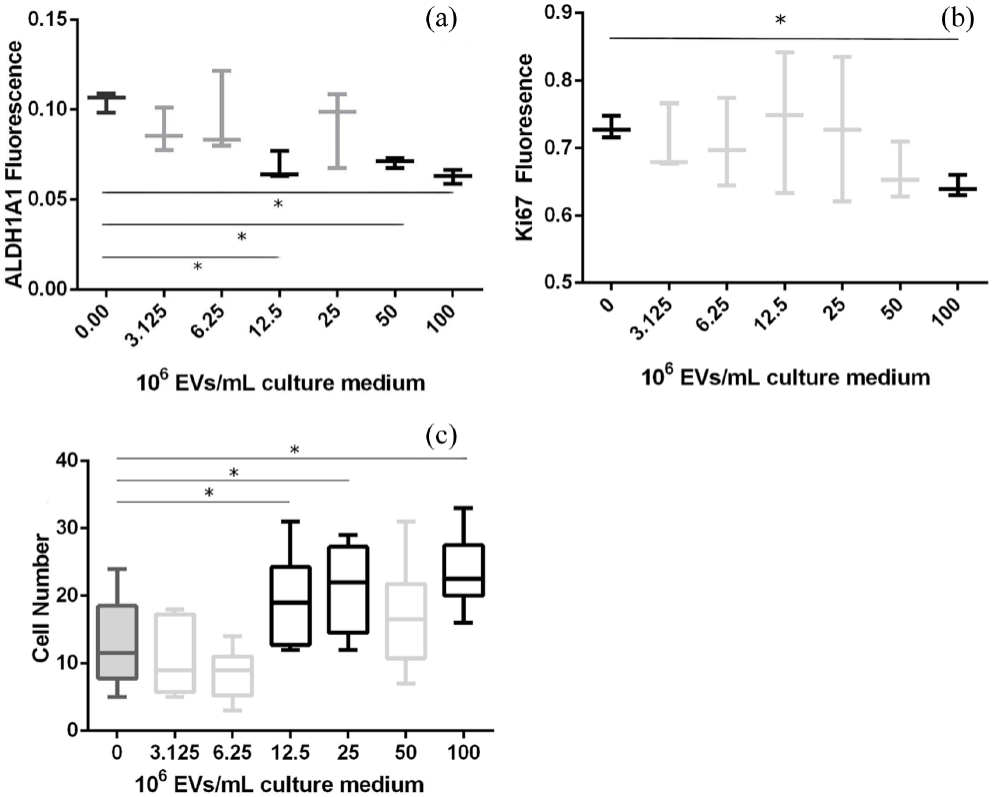
MCF7 cells treated with a dilution series of purified MSC-derived EVs for 24 h before being probed for (a) the cancer stem cell marker, ALDH1A1, and (b) the proliferation marker, Ki67 (relative fluorescence calculated via normalisation to CellTag 700; n = 3). (c) MCF7 adhesion assay following pre-treatment with MSC-derived EVs for 24 h (10 fields from 3 wells; 20× magnification). Asterisks denote p < 0.005 versus control condition.

It has previously been described that MSCs possess the capacity for tumour growth and suppression.^26,27^ Therefore, to further study the effect of MSC-derived EVs upon MCF7 cells, cell proliferation, stem cell-like phenotype and cell adhesion were assessed. ALDH1A1 is a protein that is present in malignant BCCs which have undergone EMT^28^ and as such is an established cancer stem cell marker;^29,30^ a lower ALDH1A1 expression correlates with a less active, stem cell-like cell. Here, following exposure to purified MSC-derived EVs, MCF7 cells demonstrated a decrease in ALDH1A1 (Figure 4(a)), indicating that the cells are exhibiting a less active/tumourigenic cell phenotype.^31,32^ Indeed, ALDH1A1 levels decreased with increased EV concentration, suggesting an inverse correlation.

Confirmation of this lower tumourigenicity was achieved via quantification of Ki67 protein levels. Ki67 is a protein that is present in all stages of cell cycle except G_0_ and is strongly associated with tumour cell proliferation and growth; clinically, it has been shown to correlate with metastasis and the clinical stage of tumours.^33,34^ In this study, exposure to MSC-derived EVs caused a significant decrease (p < 0.005) in Ki67 expression in MCF7 cells (Figure 4(b)); again, an inverse correlation was noted. This suggests that MCF7 cells have reduced proliferation and may have initiated a switch to a dormant state in response to the MSC EVs. MCF7 cells cultured with MSC EVs in monolayer were assayed for viability over 5 days. No significant change in cell viability was seen (supplementary Figure 2).

An increased cell adhesion is key to an epithelial cell phenotype. Therefore, in addition to probing for intracellular protein markers, MCF7 monolayers were cultured with MSC-derived EVs for 24 h and subsequent cell adhesion potential was assessed. A reciprocal relationship was observed, with a significant increase in MCF7 adhesion alongside increasing MSC-derived EV concentration (Figure 4(c)). Collectively, this correlates with a decrease in ALDH1A1 and proliferation, with the MSC-derived EVs reinforcing an epithelial cell phenotype. Expression of EMT marker genes was analysed in MCF7 spheroids cultured in the presence or absence of MSC spheroids to establish the effect of MSC paracrine signalling, including EVs, on MCF7 EMT. Results indicated that MCF7 spheroids co-cultured with MSC spheroids had an increase in the EMT markers e-cadherin and keratin19, with a reduction in mesenchymal markers vimentin and JUP. This suggests that MSCs encouraged a transition to the epithelial state (Supplementary Figure 3).

## Conclusion

BCC metastasis into the bone marrow niche involves a complex series of paracrine signalling and cell-cell interactions. However, here we show that treating MCF7 cells with just MSC-derived EVs appears to initiate an epithelial cell phenotype with potential dormancy. This suggests that MSC-derived EVs contribute to the net loss in tumourigenicity of invading BCCs, allowing them to engraft within the marrow in a cycling quiescent state, ultimately resulting in anti-cancer drug resistance. The two peaks seen in Figure 1 indicate a mixed population of both exosomes and larger microvesicles; these are trafficked out of the cell in different ways and as such their contents may also be different. While there is some evidence in support of the role of microRNAs in initiating dormancy,^4,21^ further research into the cargo of both populations of EVs is necessary, namely which proteins and small molecules/metabolites may be involved in BCC dormancy.

## Supplemental Material

J_Casson_Supplementary_data – Supplemental material for Mesenchymal stem cell-derived extracellular vesicles may promote breast cancer cell dormancy Click here for additional data file. Supplemental material, J_Casson_Supplementary_data for Mesenchymal stem cell-derived extracellular vesicles may promote breast cancer cell dormancy by Jake Casson, Owen G Davies, Carol-Anne Smith, Matthew J Dalby and Catherine C Berry in Journal of Tissue Engineering

## References

[1] Cancer Research UK. Breast cancer statistics, 2016, https://www.cancerresearchuk.org/health-professional/cancer-statistics/statistics-by-cancer-type/breast-cancer#heading-Zero (accessed 2 July 2018).

[2] Hüsemann Y Geigl JB Schubert F et al Systemic spread is an early step in breast cancer. Cancer Cell 2008; 13(1): 58–68. 18167340 10.1016/j.ccr.2007.12.003

[3] American Cancer Society. Bone metastasis key statistics, 2014, http://www.cancer.org/treatment/understandingyourdiagnosis/bonemetastasis/bone-metastasis-key-statistics1 (accessed 7 April 2018).

[4] Bliss SA Sinha G Sandiford O et al Mesenchymal stem cell-derived exosomes stimulates cycling quiescence and early breast cancer dormancy in bone marrow. Cancer Res 2016; 76: 1092. 10.1158/0008-5472.CAN-16-1092 27569215

[5] Diel IJ Kaufmann M Goerner R et al Detection of tumor cells in bone marrow of patients with primary breast cancer: a prognostic factor for distant metastasis. J Clin Oncol 1992; 10(10): 1534–1539. 1403032 10.1200/JCO.1992.10.10.1534

[6] Liu F Gu LN Shan BE et al Biomarkers for EMT and MET in breast cancer: an update. Oncol Lett 2016; 12(6): 4869–4876. 28105194 10.3892/ol.2016.5369 PMC5228449

[7] Yeung KT Yang J. Epithelial-mesenchymal transition in tumor metastasis. Mol Oncol 2017; 11(1): 28–39. 28085222 10.1002/1878-0261.12017 PMC5242415

[8] Tsai JH Yang J. Epithelial-mesenchymal plasticity in carcinoma metastasis. Gene Dev 2013; 27(20): 2192–2206. 24142872 10.1101/gad.225334.113 PMC3814640

[9] Walker ND Patel J Munoz JL et al The bone marrow niche in support of breast cancer dormancy. Cancer Lett 2016; 380(1): 263–271. 26546045 10.1016/j.canlet.2015.10.033

[10] Psaila B Lyden D. The metastatic niche: adapting the foreign soil. Nat Rev Cancer 2009; 9(4): 285. 19308068 10.1038/nrc2621 PMC3682494

[11] Khoon MCS Experimental models of bone metastasis: opportunities for the study of cancer dormancy. Adv Drug Deliver Rev 2015; 94: 141–150. 10.1016/j.addr.2014.12.007 25572003

[12] Zhang XHF Giuliano M Trivedi MV et al Metastasis dormancy in estrogen receptor-positive breast cancer. Clin Cancer Res 2013; 19(23): 6389–6397. 24298069 10.1158/1078-0432.CCR-13-0838 PMC3878717

[13] Luker KE Luker GD. Functions of CXCL12 and CXCR4 in breast cancer. Cancer Lett 2016; 238(1): 30–41. 10.1016/j.canlet.2005.06.021 16046252

[14] Liu S Ginestier C Ou SJ et al Breast cancer stem cells are regulated by mesenchymal stem cells through cytokine networks. Cancer Res 2011; 71(2): 614–624. 21224357 10.1158/0008-5472.CAN-10-0538 PMC3100554

[15] Casson J O’Kane S Smith CA et al Interleukin 6 plays a role in the migration of magnetically levitated mesenchymal stem cells spheroids. Appl Sci 2018; 8(3): 412.

[16] Davies OG Cox SC Williams RL et al Annexin-enriched osteoblast-derived vesicles act as an extracellular site of mineral nucleation within developing stem cell cultures. Sci Rep 2017; 7(1): 12639. 10.1038/s41598-017-13027-6 PMC5626761 28974747

[17] Robbins PD Morelli AE. Regulation of immune responses by extracellular vesicles. Nat Rev Immunol 2014; 14(3): 195. 10.1038/nri3622 PMC4350779 24566916

[18] Yáñez-Mó M Siljander PRM Andreu Z et al Biological properties of extracellular vesicles and their physiological functions. J Extracell Vesicles 2015; 4(1): 27066. 10.3402/jev.v4.27066 PMC4433489 25979354

[19] Wu J Qu Z Fei ZW et al Role of stem cell-derived exosomes in cancer. Oncol Lett 2017; 13(5): 2855–2866. 28521391 10.3892/ol.2017.5824 PMC5431232

[20] Ono M Kosaka N Tominaga N et al Exosomes from bone marrow mesenchymal stem cells contain a microRNA that promotes dormancy in metastatic breast cancer cells. Sci Signal 2014; 7(332): ra63. 24985346 10.1126/scisignal.2005231

[21] Vallabhaneni KC Penfornis P Dhule S et al Extracellular vesicles from bone marrow mesenchymal stem/stromal cells transport tumor regulatory microRNA, proteins, and metabolites. Oncotarget 2015; 6(7): 4953. 10.18632/oncotarget.3211 PMC4467126 25669974

[22] Del Fattore A Luciano R Saracino R et al Differential effects of extracellular vesicles secreted by mesenchymal stem cells from different sources on glioblastoma cells. Expert Opin Biol Ther 2015; 15(4): 495–504. 25539575 10.1517/14712598.2015.997706

[23] Salomon C Ryan J Sobrevia L et al Exosomal signaling during hypoxia mediates microvascular endothelial cell migration and vasculogenesis. PLoS ONE 2013; 8(7): e68451. 10.1371/journal.pone.0068451 PMC3704530 23861904

[24] Gong L Bao Q Hu C et al Exosomal miR-675 from metastatic osteosarcoma promotes cell migration and invasion by targeting CALN1. Biochem Biophys Res Commun 2018; 500(2): 170–176. 29626470 10.1016/j.bbrc.2018.04.016

[25] Lewis EEL Wheadon H Lewis N et al A quiescent, regeneration-responsive tissue engineered mesenchymal stem cell bone marrow niche model via magnetic levitation. ACS Nano 2016; 10(9): 8346–8354. 27602872 10.1021/acsnano.6b02841

[26] Lee JK Park SR Jung BK et al Exosomes derived from mesenchymal stem cells suppress angiogenesis by down-regulating VEGF expression in breast cancer cells. PLoS ONE 2013; 8(12): e84256. 10.1371/journal.pone.0084256 PMC3877259 24391924

[27] Li W Ma H Zhang J et al Unraveling the roles of CD44/CD24 and ALDH1 as cancer stem cell markers in tumorigenesis and metastasis. Sci Rep 2017; 7(1): 13856. 10.1038/s41598-017-14364-2 PMC5653849 29062075

[28] Vazquez-Santillan K Melendez-Zajgla J Jimenez-Hernandez LE et al NF-kappaΒ-inducing kinase regulates stem cell phenotype in breast cancer. Sci Rep 2016; 6: 37340. 10.1038/srep37340 PMC5120353 27876836

[29] Marcato P Dean CA Pan D et al Aldehyde dehydrogenase activity of breast cancer stem cells is primarily due to isoform ALDH1A3 and its expression is predictive of metastasis. Stem Cells 2011; 29(1): 32–45. 21280157 10.1002/stem.563

[30] Ma F Li H Li Y et al Aldehyde dehydrogenase 1 (ALDH1) expression is an independent prognostic factor in triple negative breast cancer (TNBC). Medicine 2017; 96(14): e6561. 10.1097/MD.0000000000006561 PMC5411217 28383433

[31] Tomita H Tanaka K Tanaka T et al Aldehyde dehydrogenase 1A1 in stem cells and cancer. Oncotarget 2016; 7(10): 11018. 10.18632/oncotarget.6920 PMC4905455 26783961

[32] Li LT Jiang G Chen Q et al Ki67 is a promising molecular target in the diagnosis of cancer. Mol Med Rep 2015; 11(3): 1566–1572. 25384676 10.3892/mmr.2014.2914

[33] Scholzen T Gerdes J. The Ki-67 protein: from the known and the unknown. J Cell Physiol 2000; 182(3): 311–322. 10653597 10.1002/(SICI)1097-4652(200003)182:3<311::AID-JCP1>3.0.CO;2-9

[34] Lin R Wang S Zhao RC. Exosomes from human adipose-derived mesenchymal stem cells promote migration through Wnt signaling pathway in a breast cancer cell model. Mol Cell Biochem 2013; 383(1–2): 13–20. 23812844 10.1007/s11010-013-1746-z

