# Supplementary material for: Mesenchymal stem cell-derived extracellular vesicles may promote breast cancer cell dormancy
Source: J Tissue Eng. 2018 Dec 25;9:2041731418810093. doi: 10.1177/2041731418810093 (PMC6311537; doi:10.1177/2041731418810093)
Supplement: J_Casson_Supplementary_data – Supplemental material for Mesenchymal stem cell-derived extracellular vesicles may promote breast cancer cell dormancy [file J_Casson_Supplementary_data.pdf]

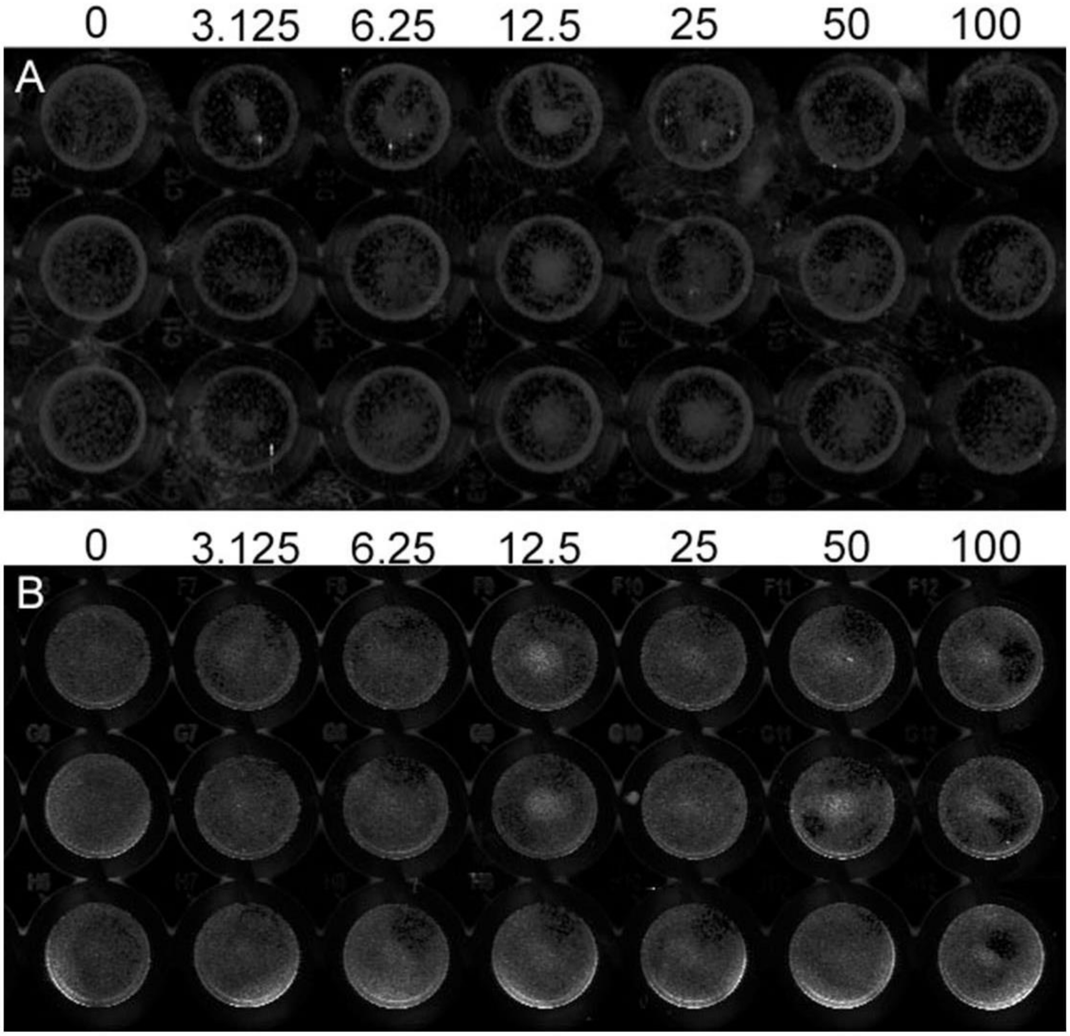

C

Normalised to Celltag		EV/mL x10^6					
	0	3.125	6.25	12.5	25	50	100
Ki67	0.7152	0.76599	0.77423	0.84144	0.83476	0.70941	0.66031
	0.74703	0.67696	0.69645	0.74879	0.72646	0.6273	0.63864
	0.72661	0.67895	0.64407	0.63234	0.62048	0.65263	0.62987
ALDH1A1	0.09817	0.08556	0.12146	0.0641	0.09863	0.07134	0.06294
	0.10898	0.07743	0.08335	0.06291	0.10848	0.07297	0.05873
	0.10674	0.1013	0.08011	0.07692	0.06743	0.06739	0.06655

Supplementary figure 1. In cell western assay of MCF7 cells treated with a dilution series of purified MSC-derived EVs for 24 hours before being probed for (A) the proliferation marker, Ki67, and (B) the cancer stem cell marker, ALDH1A1. Red indicates CellTag 700, green indicates Ki67 or ALDH1A1 respectively (relative fluorescence calculated *via* normalisation to CellTag 700; n=3, concentration of EVs x 10^6). (C) Associated data for Ki67 or ALDH1A1 fluorescence normalised to CellTag fluorescence.

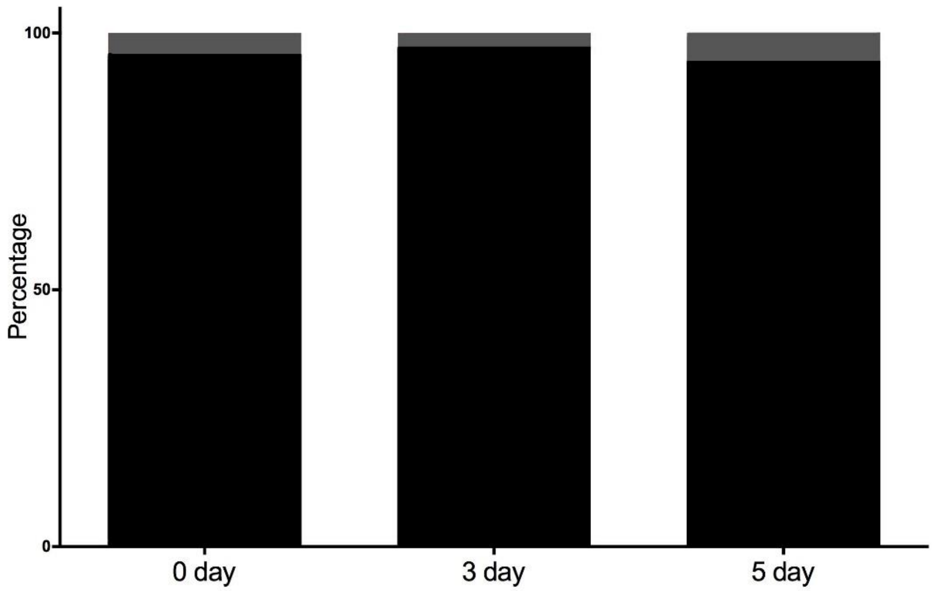

Supplementary figure 2. Live/dead assay of MCF7 cells cultured with 10^8/mL MSC EVs. Calcein AM fluorescence indicated by blue shows live cells, where ethidium fluorescence indicated by red shows dead cells. Imaged using Zeiss Axio Vert A1 microscope at 20x magnification; n=6

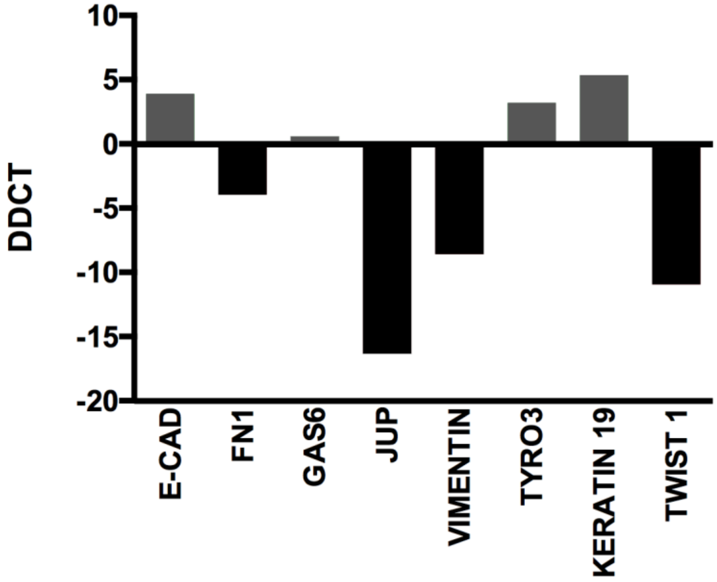

Supplementary figure 3. Analysis of expression of EMT-associated genes in MCF7 spheroids cultured within a 3D collagen gel in the presence of MSC spheroids normalised to MCF7 spheroids cultured alone. This graph shows the fold change in gene expression, identified by Delta-Delta-Ct (DDCT) analysis (n=9).
